# The various facets of orthorexic eating behavior: five case reports of individuals with supposed orthorexia nervosa

**DOI:** 10.1186/s40337-024-00988-z

**Published:** 2024-02-21

**Authors:** Friederike Barthels, Maren Fischer, Raphaela Keskini, Amelie Marie Schöl, Reinhard Pietrowsky

**Affiliations:** Institute of Experimental Psychology, Department of Clinical Psychology, Heinrich Heine University Düsseldorf, Universitätsstraße 1, 40225 Düsseldorf, Germany

**Keywords:** Orthorexia nervosa, Eating disorders, Anorexia nervosa, Healthy eating, Illness anxiety, Case report

## Abstract

**Background:**

Orthorexia nervosa, defined as a fixation on eating healthy according to subjective criteria, is recently being discussed as another variant of disordered eating behavior. Further characteristics are rigid adherence to nutritional rules, anxieties and avoidance behavior in the context of eating as well as a focus on health, not on body shape or weight loss, which is supposed to differentiate orthorexic from other disordered eating behavior. Although diagnostic criteria have been suggested, they have rarely been used in case reports published to date. Hence, the aim of this study was to present five individuals with supposed orthorexia nervosa, using preliminary diagnostic criteria to assess their eating behavior.

**Case presentation:**

The five cases (three females, two males) reveal the great variety of disordered eating behavior. Fear of unhealthy overweight (case A), supposed orthorexic eating behavior as a coping strategy for anorexia nervosa (case B), the exclusive consumption of animal products with a total exclusion of fruits and vegetables (case C), the fixation on exercise and athletic goals (case D) as well as a focus on a vegan diet and unprocessed foods (case E) are facets of orthorexia nervosa with varying degrees of impairment.

**Conclusions:**

It is concluded that orthorexia nervosa manifests itself in very different ways and that more research is needed in order to determine whether it could be a useful additional category of disordered eating behavior.

## Background

Orthorexic eating behavior, described as a fixation on only eating foods considered healthy according to subjective criteria [1, 2], is recently being discussed as another variant of disordered eating behavior [3]. In contrast to the established eating disorders anorexia nervosa, bulimia nervosa and binge-eating-disorder, orthorexic symptoms and thoughts do not evolve from worries about the quantity, but from worries about the quality of food [1, 4]. Moreover, it is believed to contrast with the avoidant/restrictive food intake disorder (ARFID) in terms of symptomatology [5] and other aspects such as the presence of traumatic events in the context of eating (e.g. choking, vomiting) and a general lack of interest in food in ARFID [4]. It is still under debate whether orthorexia is a distinct psychopathological behavior, with discussions ranging from considering it a lifestyle phenomenon [6], seeing it as a behavioral condition, hardly distinguishable from other patterns of disordered eating behavior with very little clinical relevance [7], to assuming it to be a distinct variant of disordered eating behavior [3]. Regarding its possible nosological classification, there is slightly more research suggesting that orthorexia belongs to the spectrum of eating disorders (e.g. [8, 9]) than to the obsessive–compulsive spectrum (e.g. [10]). Additionally, individuals with more pronounced orthorexic eating behavior were found to display more symptoms associated with poor physical health [11] as well as an intensified self-observation and more illness anxiety related thoughts [12], suggesting a relationship with somatic symptom disorders.

According to Cena et al. [13], and based on an analysis of definitions in papers published until 2018, orthorexia nervosa can be described as having intense concerns about healthy eating, leading to narrowed interests and focused attention on one’s eating behavior (concern/preoccupation), eventually evolving to persistent and disturbing thoughts (obsession) and stereotyped behavior (fixation). To date, several proposals of diagnostic criteria have been published (for a review, see [13]) with the most recent one stemming from the Orthorexia Nervosa Task Force [14]. Ninety-three percent of the participating experts agreed upon the following definition of orthorexia nervosa: “Orthorexia nervosa is characterized by a strong preoccupation with one's eating behavior and with self-imposed rigid and inflexible rules which are strictly controlled and include spending an excessive amount of time planning, obtaining, preparing and/or eating one’s food”. The proposed and agreed upon diagnostic criteria provide further information on its definition, on aspects that are frequently present in individuals with ON, on duration and onset. Additionally, consequences, exclusion criteria, associated risk factors and information on differential diagnoses are provided, serving as a scientific basis for more elaborated clinical research on orthorexia. However, since these criteria were established and published after the beginning of this study, we used the proposed diagnostic criteria by Barthels, Meyer and Pietrowsky [15].

To date, research on orthorexia has focused on the development of questionnaires with varying degrees of psychometrically soundness [16, 17], mainly used to assess prevalence rates (e.g. [18, 19]), risk factors [20] and to compare levels of orthorexic eating behavior in different groups within the general population depending on their characteristics, e.g. individuals with vegetarian and vegan eating behavior (e.g. [21]) or individuals with varying levels of physical training (e.g. [22]). Another way to learn more about orthorexic eating behavior and especially about the nature of symptoms and potentially resulting distress is to analyze case reports.

Apart from Bratman and Knight’s book [1], which provides a collection of anecdotal case reports, there is a small number of case reports published in scientific journals. In one early case report published by Alén [23], a 28-year-old female is described, who refused to eat anything else than seeds because she believed this would be the most natural food group. She was severely underweight, but denied to intend weight loss. Barthels and Pietrowsky [24] described a 26-year-old female who planned her mealtimes and the composition of her meals according to a strict schedule. She did not exclude that many foods from her diet, but did worry a lot about the “correct” combination of her foods and described herself as being rather obsessive regarding her eating behavior. In 2015, Moroze et al. [25] described a 28-year-old male with a 3-year history of poor nutritional intake. He worried about the purity of the food of his diet and about his intake of micronutrients, which is why he made his own protein shakes including only pure amino acid powders. Lopes, Melo and Dias [26] described an 18-year-old female with comorbid depression who spent several hours a day researching on healthy nutrition and preparing her meals. She excluded many foods from her diet, which led to weight loss, and reported having conflicts with her family and friends due to her eating habits. This case report is, according to our knowledge, the first and only one also providing information on conducted treatment. The comorbid depression was treated with mirtazapine, eventually also leading to a remission of the disordered eating behavior. Furthermore, Rania et al. [27] provide descriptions of four females who were admitted to an outpatient clinic for the treatment of eating disorders and whose supposed orthorexic features where assessed using the criteria by Dunn and Bratmann [4]. Each female is described as having a different history of mental illnesses (obsessive–compulsive disorder, anorexia nervosa, illness anxiety and paranoid personality traits), resulting in different variants of supposed orthorexic behavior. Other descriptions of individuals with orthorexic eating behavior were provided by Valente et al. [28] and McGovern, Gaffney and Trimble [29] who published reports of individuals who recovered from orthorexia and of individuals who self-identified as orthorexic. However, only a few of these case reports provide information on the extent to which affected individuals meet preliminary diagnostic criteria, and on the individual’s psychopathological characteristics regarding other aspects of (disordered) eating behavior.

In the light of several studies suggesting that individuals with possible orthorexic eating behavior seek out professionals in various areas of the health care system [30–35], it seems important to provide more detailed case examples in order to inform health care professionals and researchers about possible manifestations of orthorexic eating behavior. Even if orthorexia does not prove to be a mental disorder of clinical relevance, case reports illustrating orthorexic eating behavior in all its facets could contribute to a better understanding of individuals with deviant eating behavior, of orthorexia’s demarcation from established eating disorders and whether these cases are of clinical relevance or not.

Hence, the aim of this study was to present five case reports of individuals with supposed orthorexia nervosa, that were collected in a larger study investigating orthorexic eating behavior, implicit attitudes and cognitive flexibility [36]. After verification of the presence of orthorexic eating behavior using the Düsseldorf Orthorexia Scale [37] and preliminary diagnostic criteria [15], five case examples from those who agreed to have their cases published were selected based on the criterion to present examples displaying different facets of orthorexia nervosa. In addition to a narrative description of the supposed orthorexic symptoms and behaviors, a brief overview of sociodemographic information and different aspects of psychopathological characteristics are given in order to allow an overview of different manifestations of orthorexia nervosa.

## Methods

### Procedure

The case reports were collected in a larger study on orthorexic eating behavior, implicit attitudes and cognitive flexibility [36], which started in March 2021. Individuals with supposed orthorexia nervosa and healthy controls were recruited via social media and advertisements in universities, supermarkets, organic stores, and gyms, after obtaining ethical approval from the Ethics Committee for Non-Invasive Research Involving Humans of the Faculty of Mathematics and Natural Sciences of the Heinrich Heine University (Düsseldorf, Germany, No. BA02-2021-01). All participants gave written informed consent before participating in the study.

First, individuals filled in a screening questionnaire, which assessed among other aspects their orthorexic eating behavior using the Düsseldorf Orthorexia Scale (DOS, [37]). Individuals exceeding 25 points out of 40 were preliminarily included in the study. The clinical interview followed a few days to weeks later, investigating the participant’s eating behavior and finding out whether they fulfilled the preliminary diagnostic criteria established by Barthels, Meyer and Pietrowsky [15]. Only those individuals who fulfilled the criteria and therefore, were considered as displaying orthorexia nervosa, were assigned to the sample with supposed orthorexia nervosa and participated in the rest of the experiment, where the participants filled in the DOS a second time. When these participants with supposed orthorexic eating behavior agreed to have their anonymized data published in a case report, they were asked to give their written informed consent. Therefore, the notes taken during the interview and the data assessed were given to the participants to provide them with a good basis for their decision. Fifteen of the 29 individuals gave their consent. In order to demonstrate the variety of orthorexia nervosa, five cases with different facets of orthorexic eating behavior were selected from these 15 cases.

### Sample

In Table 1, descriptive data of the five cases are displayed. The three females and two males were between 20 and 53 years old with a mean age of 28.2 (*SD* = 14.2) years. Their body-mass-index (BMI) varied between 15.0 and 26.5 kg/m^2^, with a mean of 22.3 (*SD* = 4.4) kg/m^2^.

**Table 1:** Sociodemographic characteristics of the presented cases

Case	A	B	C	D	E
Age (in years)	27	20	53	20	21
Gender	Female	Female	Male	Male	Female
BMI kg/m^2^	26.5	15.1	24.2	24.4	21.3
Eating habits*	Not specified	Vegan	Fasting	Vegetarian	Partly vegetarian, diet for weight loss
DOS score T1	30	33	40	27	35
DOS score T2	32	33	40	26	33

BMI, Body-Mass-Index; DOS, Düsseldorf Orthorexia Scale. *Self-description of the participant; T1, DOS score assessed at the time of the participant’s study registration; T2, DOS score assessed a few days to weeks later in the interview on the participant’s eating behavior

The DOS score assessed at the time of the participant’s study registration varied between 27 and 40, with a mean of 33.0 (*SD* = 4.4) points. The DOS score assessed a few days to weeks later during the interview regarding the participant’s eating behavior varied between 26 and 40, with a mean of 32.8 (*SD* = 5.0) points.

### Material

A questionnaire on sociodemographic aspects was used in order to assess the participants’ age, gender, height and weight, and their general eating habits (vegetarian, vegan, restrictions due to allergies, fasting, diet for weight loss etc.).

The Düsseldorf Orthorexia Scale [37] was used to assess orthorexic eating behavior. The DOS contains of 10 items which are to be answered on a 4-point-scale from 1 (*does not apply to me*) to 4 (*applies to me),* with high scores indicating orthorexic eating behavior. With its satisfactory reliability [16] and indications of construct validity [38], the DOS is a promising tool to assess orthorexic eating behavior. In this study, the suggested lower preliminary cut-off of 25 points was used in order to also include individuals in the study who were at risk of displaying orthorexic eating behavior. In the whole sample, including individuals with and without supposed orthorexia nervosa (preliminary n = 69, [36]), Cronbach’s Alpha of the DOS was 0.954 in the screening questionnaire and 0.949 in the interview session.

The Eating Disorder Inventory-2 (EDI-2; 39) was used to assess psychopathological characteristics associated with eating disorders. The 91 items are divided into 11 subscales (see Table 3) and are to be rated on a 6-point scale from 1 (*never*) to 6 (*always*), with higher levels indicating a tendency towards the presence of symptoms associated with eating disorders. The reliability of most subscales is satisfactory and there is also evidence of construct validity. In the whole sample, including individuals with and without supposed orthorexia nervosa (preliminary n = 69, [36]), Cronbach’s Alpha of the EDI-2 was 0.969.

The German version of the Intuitive Eating Scale-2 (IES-2; 40) was used to assess intuitive eating behavior, which was developed to measure “an adaptive eating behavior that is characterized by eating in response to physiological hunger and satiety cues, rather than situational and emotional stimuli” [40]. It contains of 23 items on four scales (see Table 4) which are to be answered on a 5-point scale reaching from 1 (*strongly disagree*) to 5 (*strongly agree*). The IES-2 is reported to have satisfactory psychometric properties. In the whole sample, including individuals with and without supposed orthorexia nervosa (preliminary n = 69, [36]), Cronbach’s Alpha of the IES-2 was 0.902.

Using the preliminary diagnostic criteria proposed by Barthels, Meyer and Pietrowsky [15], a semi-structured interview was designed in order to assess whether the examined individuals displayed orthorexic eating behavior. Ten questions were developed to reflect the seven main and sub criteria (see Table 2) and allowed the investigator to evaluate in how far the individual’s described behavior fulfilled the preliminary criteria. In order to include the individual in the sample with supposed orthorexic eating behavior, the answers to at least seven out of these ten questions had to indicate orthorexic eating behavior. Furthermore, care was taken to ensure that the pattern of answered questions indicating orthorexic eating behavior was consistent with the original recommendation of the preliminary diagnostic criteria. According to Barthels, Meyer and Pietrowsky [15], criteria A, B, C and E must be clearly fulfilled. Criterion D should be fulfilled at least partially. The question corresponding to criterion E was used to exclude participants from the study whose sole focus was weight loss. Hence, it was not included in the 10 questions assessing orthorexic eating behavior. In the original recommendation, diagnosing atypical anorexia nervosa was recommended when criterion E was not clearly fulfilled. In order to fully assess the criteria, additional questions were asked for clarification, if needed. The interview usually lasted about 45 to 60 min, but in individual cases it could take up to two hours.

**Table 2:** Diagnostic criteria according to Barthels, Meyer and Pietrowsky [15] and the corresponding questions used in the interview to evaluate the displayed orthorexic eating behavior

	Criterion	Interview questions
A	Enduring and intensive preoccupation with healthy nutrition, healthy food and healthy eating	(1) How much time do you spend on an average day with your nutrition (including planning, purchasing, preparing, research etc.)?
		(2) Do you neglect your daily duties due to your preoccupation with your eating behavior? Or do you have difficulties concentrating on them?
B	Pronounced anxieties about food as well as extensive avoidance of foods considered unhealthy according to subjective beliefs	(3) Are there any foods you are afraid of because you consider them to be really unhealthy?
		(4) Do you have any strategies that help you to avoid these foods?
C(1)	At least two overvalued ideas concerning the effectiveness and potential health benefits of foods	(5) What do you think that your healthy nutrition does to your body?
	AND/OR	
C(2)	Ritualized preoccupation with buying, preparing and consuming foods, which is not due to culinary reasons but stems from overvalued ideas. Deviation or impossibility to adhere to nutrition rules causes intensive fears, which can be avoided by a rigid adherence to the rules	(6) Do you have established nutritional rules?
		(7) Do you have any rituals, e.g. regarding the preparation or the purchasing of your foods, which you use to make your food even healthier?
D(1)	The fixation on healthy eating causes suffering or impairments of clinical relevance in social, occupational or other important areas of life and/or negatively affects children (e. g. feeding children in an age-inappropriate way)	(8) Has your diet ever led to problems or arguments with friends/family?
		(9) To what extent do you feel affected by your diet?
	AND/OR	
D(2)	Deficiency syndrome due to disordered eating behavior. Insight into the illness is not necessary, in some cases the lack of insight might be an indicator for the severity of the disorder	(10) Have you ever been diagnosed with nutrient deficiencies, malnutrition or obesity?
E	Intended weight loss and underweight may be present, but worries about weight and shape do not dominate the syndrome	What is the primary purpose of your diet? (promote health, prevent illness, reduce weight)*

*If participants answered that the purpose of their diet was only to lose weight, there were excluded from the orthorexic group. Hence, this question was used to assess an exclusion criterion and was not among the 10 questions used to assess orthorexic eating behavior during the interview

Additionally, several general questions on the participant’s eating behavior and adjacent areas were asked in order to get a good overview of the individual’s eating behavior and also to be able to evaluate the criteria in a larger context. Some of these questions were: “How would you describe your eating behavior?”, “What do you pay special attention to in your diet?”, “Are there any substances, foods or food groups that you do not want to have in your diet (e.g. additives, sugar, fat, low-carb, flavor enhancers etc.)?” and “Do you exercise?”. The following two questions, created on the basis of formulations used in various diagnostic clinical interviews, were used in order to demarcate the displayed behavior from obsessive–compulsive disorder and delusional disorders: “Do you sometimes have disturbing thoughts that come into your mind against your own will?”, “Have you ever had the feeling of having to eat very strictly in a certain way, although you actually feel it is not meaningful?” These questions as well as the questions regarding the criteria were sorted and asked in a way that allowed a more natural conversation about the participant's eating behavior. All questionnaires were used in their German versions and also the interview was conducted in German.

### Analysis

The sum scores of the questionnaires were calculated according to the instructions in the corresponding manuals. The body mass index was calculated using the following formula: weight in kg/height in cm squared. Individual mean scores (*S*) for the scales and subscales as well as corresponding percentiles (*P*) are reported. Additionally, the range of reachable scores is reported in order to allow a classification of scores when percentiles were not published. For the description of the sample, group means (*M*) and standard deviations (*SD*) were calculated using Microsoft Excel and are reported. The description of the cases follows a narrative design. Since there is no commonly agreed on severity assessment of orthorexia nervosa, the number and intensity of fulfilled diagnostic criteria was used as a rough approximation. Due to the descriptive design of the study, no inference statistics were calculated.

## Case presentations

### Descriptive data

For each case, the individual scores on the questionnaires’ subscales are presented in Tables 3 and 4.

Table 3 reveals that Ms. A, Mr. C and Ms. E displayed high scores on most of the EDI-2’s subscales. The scores of Mr. D were not elevated, whereas the scores of Ms. B were very low.

Table 4 reveals that on the subscale Unconditional Permission To Eat, all individuals displayed low levels, whereas on the subscale Eating For Physical Rather Than Emotional Reasons, all individuals reached moderate scores. On the subscale Reliance On Internal Hunger And Satiety Cues, Mr. C and Ms. E displayed slightly higher scores than the other individuals. On the subscale Body-Food Choice Congruence, nearly all individuals displayed very high scores.

**Table 3:** Scores (*S*) and percentiles (*P*), according to the corresponding female or male reference group by Paul and Thiel [39] of psychopathological characteristics of disordered eating behavior, assessed with the Eating Disorder Inventory-II

Case	A		B		C		D		E	
Subscale	*S*	*P*	*S*	*P*	*S*	*P*	*S*	*P*	*S*	*P*
Drive For Thinness	36	99	19	65	29	99	15	70	38	99
Bulimia	9	45	7	15	11	80	11	80	9	45
Body Dissatisfaction	47	95	16	10	15	25	21	50	43	90
Ineffectiveness	32	90	12	1	26	70	Missing	–	18	20
Perfectionism	34	99	14	40	23	90	22	85	29	99
Interpersonal Distrust	19	60	9	1	32	99	13	5	23	90
Interoceptive Awareness	17	20	14	5	21	65	19	50	22	55
Maturity Fears	42	99	12	1	27	95	20	45	29	99
Asceticism	21	90	14	30	22	95	14	40	34	99
Impulse Regulation	14	5	12	1	24	60	15	10	22	50
Social Insecurity	31	99	10	1	34	99	15	10	24	70

**Table 4:** Scores (*S*) and range (*R*) of intuitive eating behavior, assessed with the Intuitive Eating-Scale-2 [40]

Case	A	B	C	D	E	
Subscale	*S*	*S*	*S*	*S*	*S*	*R*
Unconditional Permission To Eat	2.17	2.00	1.00	2.83	1.83	1–6
Eating For Physical Rather Than Emotional Reasons	4.88	5.00	5.00	5.00	3.75	1–8
Reliance On Internal Hunger And Satiety Cues	3.67	3.67	4.50	2.00	4.67	1–6
Body-Food Choice Congruence	Missing	5.00	5.00	5.00	3.67	1–5

Case reports

Ms. A: Fear of unhealthy overweight^1^

Description of eating behavior

Ms. A described her eating behavior as “very healthy, very disciplined and quite monotonous”. She paid close attention to only eating unprocessed, fresh foods and to the composition of her diet. She kept track of her nutrient intake, especially of her protein intake which she has defined to be at least 1.5 g per kg of her ideal weight. Furthermore, she has determined to not eat more than 1,700 to 1,800 kilocalories per day. She has established a regular schedule for her meals and declared that it was important for her to combine foods of animal and plant origin in her meals. In total, she stated to spend about 4 to 5 h a day focusing on her eating behavior, which sometimes interfered with her daily tasks. For example, she occasionally took less care of her daily tasks, put them off, or was not fully focused when she completed them. Her main motivation for her keeping up with her healthy eating behavior was to prevent diseases and to promote her health. She was afraid of a stroke, “fatty arteries” and high blood pressure and was desperate to prevent herself from falling back into old habits and to gain weight again (she used to weigh more than 150 kg several years ago). She said that her eating behavior was often detrimental to her mental well-being, because she had to ruminate a lot about her eating behavior and that this took up very much time.

Classification according to preliminary diagnostic criteria

Ms. A's eating behavior was determined by her fear of overweight and related consequences for her health. With regard to the preliminary diagnostic criteria (see Tables 2 and 5), it can be stated that Ms. A clearly fulfilled two of these criteria and partly fulfilled three of them, while criterion C(1) was not fulfilled and there were not enough information to evaluate criterion D(2). Regarding criterion E, although Ms. A intended to lose weight, it has to be stated that her worries about her weight were clearly health-related and not shape-related. Overall, it can be said that Ms. A. supposedly displayed orthorexic eating behavior to a mild extent.

**Table 5:** Fulfilled diagnostic criteria (see Barthels, Meyer and Pietrowsky [15] of the cases

	Case	A	B	C	D	E
	*Criterion*					
A	Intensive Preoccupation	xx	xx	xx	xx	xx
B	Anxieties and avoidance	x	xx	xx	x	xx
C(1)	Overvalued ideas	–	–	xx	–	–
C(2)	Ritualization; fear when transgressing rules	xx	x	x	x	xx
D(1)	Suffering or impairments	x	xx	xx	x	x
D(2)	Nutrient deficiency	o	–	o	o	o
E	Focus on health, not on weight loss	x	x	xx	x	x
	Overall assessment of orthorexia nervosa	Mild	Moderate	Severe	Mild	Moderate

xx, clearly fulfilled; x, partially fulfilled; –, not fulfilled; o, not enough or no information

Ms. B: Supposed orthorexic eating behavior as a coping strategy for anorexia nervosa^1^

Description of eating behavior

Ms. B declared to only eat plant-based, “healthy and fresh” food. She bought organic food whenever possible and didn’t eat any processed food. Her diet included legumes, whole grains, oatmeal, ancient grains such as quinoa and buckwheat, fresh vegetables, fruit, and plenty of water. Her friends said that her diet looked "colorful and healthy" but that they could never follow it. She forwent plenty of things in her life, but it did not feel that way to her. She stated that in general, her diet was very well planned, so she didn’t even get into the situation of having to eat something unhealthy. For example, she scheduled her meal times and sometimes went home earlier, in order to stick to them. She would rather endure hunger than eat something unhealthy. Ms. B. stated that she started to focus on her diet when she was 12 years old. She also had an anorexia nervosa for several years and the focus on healthy eating had helped her to "transform nutrition into something healthy". The fact that she did not eat meat, however, is exclusively for ethical reasons. She said to feel more connected to her body and to give her body more of what it needed since eating healthily Occasionally, however, she got into trouble because of her diet, especially with her mother and with her friends, who both missed her spontaneity in the context of eating. She stated that the occupation with her eating behavior takes about 5 h per day. Hence, it sometimes interfered with her daily tasks, for example because she was unconcentrated or due to time constraints. Although she said that the routines were good for her, she occasionally missed a bit of flexibility, too, which made her feel affected by her diet quite often. This is why she would like to be more relaxed about her diet now and then. At that moment, however, the feelings of being satisfied and proud that she was able to manage her eating behavior so well dominated, so she did not want to change it.

Classification according to preliminary diagnostic criteria

Ms. B’s eating behavior was determined by overcoming her former eating disorder by now focusing on a healthy diet. With regard to the preliminary diagnostic criteria (see Tables 2 and 5), it can be stated that Ms. B fulfilled three criteria clearly and two criteria partially. Overvalued ideas (C(1)) were absent, as well as nutrient deficiencies (D(2)). Regarding the differentiation from eating disorders, she did not declare any worries about weight and shape, although she was still severely underweight. Hence, her previous anorexia nervosa seemed to be in remission, and therefore, the presence of anorexic symptoms did not speak against the simultaneous existence of orthorexic symptoms. Overall, it can be stated that Ms. B supposedly displayed orthorexic eating behavior to a moderate extent.

Mr. C: Only meat, no vegetables^1^

Description of eating behavior

Mr. C. stated that his diet consisted exclusively of animal products, like meat, fish, butter and eggs. He did not eat any fruits or vegetables. He followed a very high-fat and high-protein diet, and he considered fat to be the most important nutrient, while he completely avoided carbohydrates. He emphasized he did not consume any sugar, alcohol or caffeine. He regularly went on fasting diets, which he only interrupted if he got too thin. Apart from that, he could fast "forever" because he saw it as a "cure" in which stem cells and growth hormones were activated.

Mr. C. has been following this diet for health reasons for about 15 years. At that time, rheumatism had caused him "insane pain", which “had not interested anyone”, including his family and medical professionals. No treatment helped, which is why today he has lost all respect for conventional medicine. Only the exclusion of several foods led to an improvement of his rheumatism. He developed this diet after intensive research and found out, among other things, that some fruits and vegetables were toxic. As an example, he mentioned spinach and strawberries containing oxalate, which could lead to kidney stones. He also stated that eating fruit would give you a "non-alcoholic fatty liver" and that "anything with more than two ingredients is garbage." Furthermore, he considered claims that fat is unhealthy to be “propaganda”. Additionally, it was "extremely important" for him to exercise. He declared to walk 30 km a day, and he also cycled, did fitness and used to swim.

Currently, Mr. C. declared to feel “fit as a fiddle”. His rheumatism symptoms have disappeared, and he had "blood levels like a 16-year-old”. He always felt awake and could “talk to 10 people at once” because he was not in “that carbohydrate fog”. As soon as he deviated from his current diet, he immediately felt worse, for example, his eyes started to cause problems and he was in more pain again.

However, his diet also led to difficulties. Mr. C. felt “disgust and horror” in view of what other people eat. He recognized from their “aura” that they were “metabolically ill”. He had to go “sneaky ways” to avoid food smells in the city and also the smells of other people. As a result, he no longer had contact with his family or friends. He is sometimes dismissed as “a crank”, even by doctors, but they are “totally clueless”; he thought he was the only person who is educated in metabolic issues. Occasionally, people also made fun of him.

Classification according to preliminary diagnostic criteria

Mr. C’s eating behavior was clearly focused on health aspects in all points. A certain body weight only plays an indirect role, as he was careful not to lose too much weight during his fasting episodes. Regarding the diagnostic criteria (see Tables 2 and 5), it can be stated that Mr. C clearly fulfilled five criteria. Regarding the presence of overvalued ideas, he was the only case in this study fulfilling criterion C(1). Criterion C(2) was considered to be partially fulfilled, because he did not describe any rituals, albeit his diet is extremely rigid. Regarding criterion D(2), it could not be clearly stated whether nutrient deficiencies were present or not. While he declared to have “blood levels like a 16-year-old”, he also said that he had no trust in conventional medicine, which raises doubts about his claim to have had checked his blood levels. All in all, it can be stated that Mr. C’s supposed orthorexic eating behavior was very pronounced.

Mr. D: Healthy eating and exercise

Description of eating behavior

Mr. D described his eating behavior as very controlled and healthy. He aimed to consume about 25% of his total daily calories from proteins in order to support his scheduled exercise routine of five to six training sessions per week. He ate very regularly throughout the day to ensure a continuous intake of nutrients and he preferred unprocessed foods. For ethical reasons, he was a vegetarian, and apart from his focus on proteins, he also paid close attention to eating fruits and vegetables in every meal. He used an app to track his food intake and sometimes he entered his meals one day ahead in the app. He also kept track of his calorie intake not only to shape his body, but also to promote his health.

He was preoccupied with his eating behavior for about 2 to 3 h a day, which sometimes interfered with his daily tasks. Once, he did not turn in an assignment for university on time because he was busy figuring out "the best food" and researching the quantities of the nutrients. Although there were no foods that really scared him, he nonetheless avoided certain foods, for example fatty foods, because otherwise he would be very upset about the “wasted calories”. He claimed that usually, his diet made him feel fit and powerful, but he sometimes also experienced negative emotions due to his eating behavior. For example, eating meals that other people have cooked is an “inner struggle”, because he could not control how the meals were cooked. Furthermore, he frequently forwent indulgences in order to eat healthily. He paid close attention to stick to his schedule because he wanted to promote his health and also tried to increase his well-being and to regulate his mood with his eating behavior. This is why he almost never transgressed his nutritional rules. He rather endorsed hunger or appetite for specific foods than eat something unhealthy. However, he did not worry about getting a specific disease, he rather tried to “optimize everything”.

Classification according to preliminary diagnostic criteria

Mr. D’s eating behavior was focused on a healthy diet designed to support his athletic goals, which he did not define in the interview. With regard to the preliminary diagnostic criteria (see Tables 2 and 5), he clearly fulfilled criterion A, due to the intense focus on his eating behavior, while he partially fulfilled criteria B, C(2) and D(1). For example, he described to avoid some foods (e.g. fatty foods) but still was able to eat meals that others cooked, albeit causing him inner conflicts (criterion B). Overvalued ideas (criterion C(1)) were absent and there was not enough information to evaluate the presence of nutrient deficiencies (criterion D(2)). Regarding criterion E, it can be stated that his focus was clearly on health, however, he kept track of his calorie intake and also aimed to shape his body with his rigid exercise schedule. In this case, the ambitions to eat healthily could hardly be separated from worries about weight and shape because of the interaction with his athletic goals. However, since he did not aim to lose weight, criterion E was considered to be partially fulfilled. His supposedly orthorexic eating behavior was classified as being mild, with a strong focus on scheduled eating and exercising and with no ambitions to directly control his weight.

Ms. E: Vegan and unprocessed

Description of eating behavior

Ms. E claimed to follow a vegan diet and reported to pay close attention to a balanced eating behavior in order to promote her health and to avoid diseases. It was very important for her that her food was minimally processed or unprocessed, free from artificial additives and that it is seasonal, organic and produced locally. Furthermore, she preferred food with a low content of fat and sugar. She used to record her food intake but eventually stopped it when she noticed that it had a negative effect on her mental health. In total, she spent about 4 to 5 h a day focusing nutrition, which sometimes interfered with her daily tasks. For example, at times, she lost herself in thoughts like “Do I need to buy food? Is the shop still open? What do I need for my next meal”? which hampered her concentration. Furthermore, she experienced negative effects in her social life. Sometimes, her friends and her partner made fun of her eating habits and she reported feeling stressed by the urge “that you always have to explain yourself, for example, why you can't eat the cake someone brought to work”. Additionally, regarding her rule to only eat very small amounts of fat and no sugar, she declared to have “strong mental barriers”. She could not enjoy food with these ingredients. She also reported that her self-esteem was very strongly linked to what she ate. When she transgressed her own rules, she felt like a failure. Although she knew that in the long term, a transgression now and then would not affect her overall healthy nutrition, she felt bad in the specific moment and was also afraid of the additional calories she had taken in when eating food with fat or sugar. Sometimes, she was afraid that she might get cancer if she did not stick to her strict rules. That is why feelings of guilt and anxiety arose when she transgressed her rules and she could not understand her own weakness. Apart from eating healthily, she also paid attention to living healthily in other areas of life. For example, she did not smoke, she did not drink alcohol and she exercised on a regular basis, even though she believed that healthy eating has the greatest impact on her overall health.

She declared to have sought psychotherapeutic help in the past, due to being underweight at that time and due to her strong preoccupation with health and illness. However, in general, she was satisfied with her eating behavior and would only change it “occasionally, when I feel like it”, but she could not deny that she sometimes also felt affected by it.

Classification according to preliminary diagnostic criteria

Ms. E’s eating behavior was focused on a clean, unprocessed and vegan diet. With regard to the preliminary diagnostic criteria (see Tables 2 and 5), it can be stated that Ms. E’s eating behavior fulfilled three criteria (A, B and C(2)) clearly, because she revealed her intensive preoccupation with healthy eating in the interview, talked about several anxieties and avoidance behaviors and also of fears when transgressing her rules. Criterion D(1) and E were considered to be fulfilled partially, because she only described some aspects of impairment and also reported some minor fears regarding calorie intake. Also in this case, overvalued ideas were absent and there was not enough information to evaluate the presence of nutrient deficiencies. Overall, it can be stated that Ms. E supposedly displayed orthorexic eating behavior to a moderate extent.

## Discussion

To determine whether orthorexia should be recognized as a distinct eating disorder, not only empirical studies in large samples on the characteristic features of orthorexic eating behavior should be conducted. In order to better understand supposedly orthorexic eating behavior from the perspective of affected individuals, case studies are also needed. Since only few case reports have been published and even fewer used preliminary diagnostic criteria to determine the presence of orthorexia nervosa, the aim of this study was to present five individuals with orthorexia nervosa ascertained by using the preliminary diagnostic criteria by Barthels, Meyer and Pietrowsky [15]. The presented cases were selected from a larger sample of individuals with possible orthorexic eating behavior [36] and were not meant to provide a representative overview of orthorexic patterns, but were rather selected in terms of demonstrating a variety of different types of orthorexia.

The case of Ms. A represents the interaction of supposedly orthorexic eating behavior and weight control. However, Ms. A. did not intend to achieve a certain body shape, she rather wanted to lose weight in order to reach normal weight for health reasons. In view of her current slight overweight, the high values in the EDI-2 are not to be interpreted as indications of anorexia nervosa, but rather refer to her aim of health-related weight regulation. Having been overweight in her past, she was afraid of weight gain and diseases that are associated with overweight, which might be interpreted as a form of illness anxiety. Whereas fear of unhealthy overweight is mentioned once in the context of orthorexic eating behavior (e.g. [41]), there are quite a few studies pointing out the relationship between orthorexic eating behavior and illness anxiety [12, 27, 42]. This connection is also revealed in several cases reported by Bratman and Knight [1]. However, considering the more pronounced illness anxiety-related thoughts and fears mentioned in their cases, the connection to illness anxiety can be considered to be rather week in the case of Ms. A.

The case of Ms. B. is an example of using supposedly orthorexic eating behavior as a strategy to cope with a serious eating disorder. Ms. B’s diet was still very rigid, and given her weight, it can be assumed that her caloric intake remained quite low. However, the way she dealt with nutrition seems to be much more positive and healthier than her previous eating disorder, moving away from classic anorexic thoughts and behaviors. This is also supported by her statement that her blood results were good, despite being underweight. Since Ms. B. was undergoing psychotherapeutic treatment, it can be assumed that she had not yet overcome the eating disorder and that she will possibly succeed in loosening the restrictions more and more as the therapy progresses. Hence, this case is representative of the frequently reported interaction of orthorexia and disordered eating behavior in general (e.g. [3]) and particularly the connection with anorexia nervosa. Segura-Garcia et al. [43] and Barthels et al. [44] both published data allowing the conclusion that individuals with diagnosed anorexia nervosa might use orthorexic eating behavior as a coping strategy. Whether orthorexic eating behavior should be considered a pathological habit in these cases or whether it should rather be seen as a useful strategy to cope with potentially more life-threatening anorexic symptoms is an ethical question that needs to be discussed. At least in the case of Ms. B, however, it seemed as if she was on the road to recovery from anorexia by focusing on healthy eating, although she still needed to increase her calorie intake and to gain weight in order to achieve a healthy body weight.

The case of Mr. C is rather unique in terms of the displayed eating behavior. Frequently, orthorexic eating behavior is reported to be associated with the abandonment of meat (see e.g. [21]). However, Mr. C focussed on only eating foods from animal origin. He was the only one amongst the presented cases displaying overvalued ideas, similar to some cases reported by Bratman and Knight [1]. This case reveals that orthorexia nervosa might also include eating patterns generally not considered healthy, which could potentially lead to health issues sooner or later. Mr. C’s supposedly orthorexic eating behavior can be considered to be rather severe since it has several detrimental consequences on his mental well-being, especially on his social life. Moreover, due to his extremely restricted diet without fruits and vegetables, future nutrient deficiency symptoms cannot be ruled out. However, Mr. C did not seem to have any insight into his potentially harmful behavior; on the contrary, during the interview, he pointed out that his eating habits were superior to those of others and that he had knowledge that others did not have. Furthermore, Mr. C. very willingly gave detailed information about his eating behavior, resulting in the interview lasting more than two hours instead of the usual 45 to 60 min. Missionary zeal, which Bratman and Knight [1] mentioned in the context of orthorexia, could be inferred from this behavior as well as a certain proximity to delusional behavior.

The case of Mr. D is an example for the interaction of the fixation on healthy eating and extensive exercise behavior, which has frequently been investigated [45], suggesting a connection between these two behaviors especially in males [46]. Furthermore, he not only used healthy eating behavior to optimize his physical training, but also to regulate his mood and his mental well-being, signifying that he attached much more importance to nutrition than nurturing his body. Consequently, he did not experience many negative emotions caused by his eating behavior, nor did he report suffering frequently or to feeling impaired. Using one’s eating behavior to regulate one’s mental well-being might be considered rather dysfunctional when practiced excessively. However, the descriptions of Mr. D suggest that he displayed a mild variant of orthorexia.

The case of Ms. E is representative of the commonly reported connection of orthorexic eating behavior and vegan eating habits [47, 48]. Whereas Ms. B and Mr. D, who also pursue a vegan and a vegetarian diet respectively, both stated that they did so for ethical reasons, Ms. E has not stated any ethical reason for following a vegan diet. One study revealed that in vegans, the level of orthorexic eating behavior is linked to health and not to animal welfare [49]. Taking this result into account might suggest that in the cases of Ms. B and Mr. D, the vegan diet for ethical reasons might possibly be independent of their orthorexic eating behavior, whereas Ms. E’s vegan eating habits might be part of her aim to eat healthily. Additionally, Ms. E paid close attention to only eating unprocessed foods, which is a behavior that Bratman and Knight [1] also reported in the context of orthorexia. She seemed to be torn between following her rigid rules and sometimes transgressing them, consequently experiencing a negative mood. In general, her eating behavior was dominated by an intense preoccupation with food, anxieties and avoidance behaviors, that affected her daily and her social life as well as her ability to enjoy foods.

Regarding the diagnostic criteria, and due to the preset inclusion criteria for the sample with supposed orthorexia nervosa, it is not a surprise that every individual fulfills at least four of the seven criteria, with all of them displaying an intensive preoccupation with their eating behavior, anxieties and avoidance behavior, ritualization of eating behavior and fears when transgressing their self-imposed rules, as well as suffering or impairments in everyday life. Overvalued ideas are only present in one case (Mr. C), which is simultaneously the most severe case of orthorexia nervosa in this sample, suggesting that overvalued ideas might represent a feature which is indicative of severe orthorexic cases. However, it has to be noted that overvalued ideas, although reported in some of the cases by Bratman and Knight [1], are not present in other proposals for diagnostic criteria (for an overview, see [13]), nor are they part of the recently proposed criteria by the Orthorexia Nervosa Task Force [14].

Due to the nature of this study, it was not possible to assess and evaluate possible nutrient deficiencies, which limits the ability to evaluate criterion D(2). Regarding criterion E, which demands a focus on health, not on weight loss, operationalized by the absence of worries about weight/shape, it has to be noted that two out of the five cases (Ms. B and Mr. D) displayed worries about their weight, but not about their shape. Also, in the case of Ms. B, the interaction with her (former) diagnosis of anorexia nervosa is visible. It is still under debate whether orthorexic eating behavior is a distinct phenomenon or not [3, 7]; consequently, it is unknown whether it is necessary to have criteria for a differential diagnosis regarding weight- and shape-related eating disorders.

Nonetheless, it can be concluded with caution that these preliminary diagnostic criteria are suited to assess not only the presence, but also the level of severity of orthorexia nervosa, which is represented in the degree of fulfilment of the criteria and especially in the question whether overvalued ideas are present or not.

The association of psychopathological characteristics of eating disorders and orthorexic eating behavior that have been widely reported in literature (e.g. [5, 10]) are also reflected in the cases of this study. Three of the five cases (Ms. A, Mr. C and Ms. E) display overall high levels of symptoms associated with eating disorders, supporting the findings in literature. Since these five descriptions reveal that in all these cases a focus on healthy eating and not on weight loss and shape exist, it may be assumed that psychopathological characteristics of anorexia nervosa and bulimia nervosa might exist next to orthorexic symptoms. Another possible explanation is that the items used in the EDI-2 are not able to differentiate between the behavior that is focused on health and the behavior that is focused on weight/shape concerns. Interestingly, Ms. B, who reported having a history of anorexia nervosa, displayed surprisingly low values on the EDI-2’s subscales. Since she was receiving psychotherapeutic treatment, it is feasible that she has filled in the EDI-2 several times and has answered these questions in light of her partially recovered condition.

Regarding intuitive eating behavior, the low scores which all five individuals presented on the subscale Unconditional Permission To Eat in particular reflect orthorexic eating behavior namely not as an intuitive, but as a rule-driven and restricted eating behavior. Interestingly, nearly all individuals reached rather high scores on the subscale Body-Food Choice Congruence, indicating that the individuals followed their diets in the belief or with the intent to treat themselves. Emotional eating, assessed with the subscale Eating For Physical Rather Than Emotional Reasons does not seem to play a major role within the surveyed individuals as indicated by the mediocre value. The individuals seem to differ on the subscale Reliance On Internal Hunger And Satiety Cues, with Mr. D rather not relying on these cues, Mr. C and Ms. E rather relying on them and Ms. A and Ms. B being in the middle. Since there are only few studies on the subject of orthorexic eating behavior and intuitive eating behavior, a limited number of comparisons are possible. One study suggests that orthorexic eating behavior is associated with less positive eating behaviors, such as intuitive eating, with differences between males and females [50].

### Strengths and limitations

As far as we know, this study is the first presenting individuals with supposed orthorexia nervosa who have been evaluated using a structured interview and preliminary diagnostic criteria. Since this study started in 2021, the COVID-19 pandemic must be mentioned as a possible limitation. However, only one participant (Mr. D) reported that his eating behavior had changed due to the pandemic, namely that he ate healthier than before. All other participants answered this question in the negative. The timepoint of the onset of this study is also the reason why we were not able to use the recently published preliminary diagnostic criteria by the Orthorexia Nervosa Task Force [14]; hence, it could be seen as a limitation that we did not use the most recent proposal of diagnostic criteria, albeit both proposals show an overlap in several criteria. A further limitation which must be mentioned is that the selection of the cases is not representative. Therefore, this study does not allow any inferences about the frequency of the presented types of orthorexia nervosa.

## Conclusions

This is the first study in which individuals with supposed orthorexia nervosa were systematically interviewed. The presented cases reflect the great variety of orthorexic eating behavior ranging from carnivore to vegan, from underweight to overweight, or in combination with a focus on exercise. In addition, these cases reveal the spectrum of orthorexic symptoms, reaching from health concerns and anxieties regarding specific foods, avoidance behaviors and strict rules that impair social life to time-consuming schedules and overvalued ideas. Hence, orthorexia nervosa seems to manifest itself in a plethora of ways and there are reportedly further manifestations (e.g. the excessive intake of nutritional supplements), that were not covered in this study. As it is still unclear whether orthorexia nervosa is a distinct mental disorder or not, and in addition to case studies like the present ones, more research is needed to differentiate diverse patterns of disordered eating behavior and to determine whether it could be useful to define orthorexia nervosa as an additional category of disordered eating behavior.

## Data Availability

The data used in the current study are available from the corresponding author on reasonable request.

## Abbreviations

BMI: Body-mass-index
DOS: Düsseldorf Orthorexia Scale
EDI-2: Eating Disorder Inventory-2
IES-2: Intuitive Eating Scale-2

## Footnotes

1 For transparency, we would like to declare that more elaborated versions of these cases also appear in a German book on orthorexia nervosa: Barthels F, Pietrowsky R. Orthorektisches Ernährungsverhalten—Forschung und Praxis [Orthorexic eating behavior—Research and Practice]: Hogrefe; 2024. In order to make these cases accessible to international researchers, abbreviated versions are published in this article with permission of the publisher of the book.

## References

[1] Bratman S, Knight D (2000). Health food junkies: overcoming the obsession with healthful eating.

[2] Strahler J, Hermann A, Walter B, Stark R (2018). Orthorexia nervosa: a behavioral complex or a psychological condition?. J Behav Addict.

[3] Atchison AE, Zickgraf HF (2022). Orthorexia nervosa and eating disorder behaviors: a systematic review of the literature. Appetite.

[4] Dunn TM, Bratman S (2016). On orthorexia nervosa: a review of the literature and proposed diagnostic criteria. Eat Behav.

[5] Zickgraf HF, Ellis JM, Essayli JH (2019). Disentangling orthorexia nervosa from healthy eating and other eating disorder symptoms: Relationships with clinical impairment, comorbidity, and self-reported food choices. Appetite.

[6] Håman L, Barker-Ruchti N, Patriksson G, Lindgren E-C (2015). Orthorexia nervosa: an integrative literature review of a lifestyle syndrome. Int J Qual Stud Health Well-being.

[7] Meule A, Voderholzer U (2021). Orthorexia nervosa—It is time to think about abandoning the concept of a distinct diagnosis. Front Psychiatry.

[8] Bartel SJ, Sherry SB, Farthing GR, Stewart SH (2020). Classification of Orthorexia Nervosa: further evidence for placement within the eating disorders spectrum. Eat Behav.

[9] Hessler-Kaufmann JB, Meule A, Greetfeld M, Schlegl S, Voderholzer U (2021). Orthorexic tendencies in inpatients with mental disorders. J Psychosom Res.

[10] Zagaria A, Vacca M, Cerolini S, Ballesio A, Lombardo C (2022). Associations between orthorexia, disordered eating, and obsessive-compulsive symptoms: a systematic review and meta-analysis. Int J Eat Disorder.

[11] Oberle CD, Klare DL, Patyk KC (2019). Health beliefs, behaviors, and symptoms associated with orthorexia nervosa. Eat Weight Disord.

[12] Barthels F, Horn S, Pietrowsky R (2021). Orthorexic eating behaviour, illness anxiety and dysfunctional cognitions characteristic of somatic symptom disorders in a non-clinical sample. Eat Weight Disord.

[13] Cena H, Barthels F, Cuzzolaro M, Bratman S, Brytek-Matera A, Dunn TM (2019). Definition and diagnostic criteria for orthorexia nervosa: a narrative review of the literature. Eat Weight Disord.

[14] Donini LM, Barrada JR, Barthels F, Dunn TM, Babeau C, Brytek-Matera A (2022). A consensus document on definition and diagnostic criteria for orthorexia nervosa. Eat Weight Disord.

[15] Barthels F, Meyer F, Pietrowsky R (2015). Orthorexic eating behaviour. A new type of disordered eating. Ern Um..

[16] Meule A, Holzapfel C, Brandl B, Greetfeld M, Hessler-Kaufmann JB, Skurk T (2020). Measuring orthorexia nervosa: a comparison of four self-report questionnaires. Appetite.

[17] Opitz M-C, Newman E, Mellado ASAV, Robertson M, Sharpe H (2020). The psychometric properties of orthorexia nervosa assessment scales: a systematic review and reliability generalization. Appetite.

[18] Dunn TM, Gibbs J, Whitney N, Starosta A (2016). Prevalence of orthorexia nervosa is less than 1%: data from a US sample. Eat Weight Disord.

[19] Luck-Sikorski C, Jung F, Schlosser K, Riedel-Heller SG (2019). Is orthorexic behavior common in the general public? A large representative study in Germany. Eat Weight Disord.

[20] McComb SE, Mills JS (2019). Orthorexia nervosa: a review of psychosocial risk factors. Appetite.

[21] Brytek-Matera A (2021). Vegetarian diet and orthorexia nervosa: a review of the literature. Eat Weight Disord.

[22] Hafstad SM, Bauer J, Harris A, Pallesen S (2023). The prevalence of orthorexia in exercising populations: a systematic review and meta-analysis. J Eat Disord.

[23] Alén EM (2006). Perspectiva antropológica de un caso de ortorexia nerviosa [Anthropological perspective of a case of orthorexia nervosa]. Cult de los Cuid.

[24] Barthels F, Pietrowsky R (2012). Orthorektisches Ernährungsverhalten - Nosologie und Prävalenz [Orthorectic Eating Behaviour—Nosology and Prevalence Rates]. Psychother Psych Med.

[25] Moroze RM, Dunn TM, Holland C, Yager J, Weintraub P (2015). Microthinking about micronutrients: a case of transition from obsessions about healthy eating to near-fatal "orthorexia nervosa" and proposed diagnostic criteria. Psychosomatics.

[26] Lopes R, Melo R, Dias PB (2020). Orthorexia nervosa and comorbid depression successfully treated with mirtazapine: a case report. Eat Weight Disord.

[27] Rania M, de Filippis R, Caroleo M, Carbone E, Aloi M, Bratman S (2021). Pathways to orthorexia nervosa: a case series discussion. Eat Weight Disord.

[28] Valente M, Brenner R, Cesuroglu T, Bunders-Aelen J, Syurina EV (2020). “And it snowballed from there”: the development of orthorexia nervosa from the perspective of people who self-diagnose. Appetite.

[29] McGovern L, Gaffney M, Trimble T (2021). The experience of orthorexia from the perspective of recovered orthorexics. Eat Weight Disord.

[30] Barthels F, Gahlmann S, Schwabe T, Pietrowsky R (2021). Occurrence and relevance of orthorexic eating behaviour in clients of complementary and alternative medicine. J Public Health Epidemiol.

[31] Barthels F, Lavendel S, Müller R, Pietrowsky R (2019). Relevance of orthorexic eating behavior in nutrition counseling and nutrition therapy. Results of a nationwide survey among German nutritionists. Ern Um..

[32] Ryman FVM, Cesuroglu T, Bood Z, Syurina E (2019). Orthorexia nervosa: disorder or not? Opinions of Dutch health professionals. Front Psychol.

[33] Reynolds R, McMahon S (2019). Views of health professionals on the clinical recognition of orthorexia nervosa: a pilot study. Eat Weight Disord.

[34] Gramaglia C, Gattoni E, Ferrante D, Abbate-Daga G, Baldissera E, Calugi S (2022). What do Italian healthcare professionals think about orthorexia nervosa? Results from a multicenter survey. Eat Weight Disord.

[35] Vandereycken W (2011). Media hype, diagnostic fad or genuine disorder? Professionals' opinions about night eating syndrome, orthorexia, muscle dysmorphia, and emetophobia. Eat Disord.

[36] Barthels F, Fischer M, Keskini R, Schöl AM, Pietrowsky R. Implicit attitudes and cognitive flexibility in orthorexic individuals compared to a healthy control group. In preperation.

[37] Barthels F, Meyer F, Pietrowsky R (2015). Die Düsseldorfer orthorexie skala - konstruktion und evaluation eines fragebogens zur erfassung orthorektischen ernährungsverhaltens [duesseldorf orthorexia scale—construction and evaluation of a questionnaire measuring orthorexic eating behavior]. Z Klin Psychol Psychother.

[38] Barthels F, Amrhein J, Scharmach K, Meyer F, Pietrowsky R (2018). Konstruktvalidität der Düsseldorfer orthorexie skala: korrelationen mit der gesundheitlichen qualität des essverhaltens und essstörungsspezifischen aspekten [concurrent construct validity of the düsseldorf orthorexia scale: correlations with diet quality and specific dimensions of disordered eating behavior]. Z Klin Psychol Psychother.

[39] Paul T, Thiel A. Eating disorder inventory-2. Deutsche Version. Göttingen: Hogrefe (2005)

[40] Van Dyck Z, Herbert BM, Happ C, Kleveman GV, Vögele C (2016). German version of the intuitive eating scale: psychometric evaluation and application to an eating disordered population. Appetite.

[41] Barthels F, Meyer F, Huber T, Pietrowsky R (2017). Analyse des orthorektischen Ernährungsverhaltens von Patienten mit Essstörungen und mit Zwangsstörungen [analysis of orthorexic eating behavior in patients with eating disorder and obsessive-compulsive disorder]. Z Klin Psychol Psychother.

[42] Barthels F, Müller R, Schüth T, Friederich H-C, Pietrowsky R (2021). Orthorexic eating behavior in patients with somatoform disorders. Eat Weight Disord.

[43] Segura-Garcia C, Ramacciotti C, Rania M, Aloi M, Caroleo M, Bruni A (2015). The prevalence of orthorexia nervosa among eating disorder patients after treatment. Eat Weight Disord.

[44] Barthels F, Meyer F, Huber T, Pietrowsky R (2016). Orthorexic eating behavior as a coping strategy in patients with anorexia nervosa. Eat Weight Disord.

[45] Strahler J, Wachten H, Mueller-Alcazar A (2021). Obsessive healthy eating and orthorexic eating tendencies in sport and exercise contexts: A systematic review and meta-analysis. J Behav Addict.

[46] Strahler J, Wachten H, Stark R, Walter B (2021). Alike and different: Associations between orthorexic eating behaviors and exercise addiction. Int J Eat Disorder.

[47] Barthels F, Meyer F, Pietrowsky R (2018). Orthorexic and restrained eating behaviour in vegans, vegetarians, and individuals on a diet. Eat Weight Disord.

[48] Brytek-Matera A, Czepczor-Bernat K, Jurzak H, Kornacka M, Kołodziejczyk N (2019). Strict health-oriented eating patterns (orthorexic eating behaviours) and their connection with a vegetarian and vegan diet. Eat Weight Disord.

[49] Barthels F, Poerschke S, Müller R, Pietrowsky R (2020). Orthorexic eating behavior in vegans is linked to health, not to animal welfare. Eat Weight Disord.

[50] Rodgers RF, White M, Berry R (2021). Orthorexia nervosa, intuitive eating, and eating competence in female and male college students. Eat Weight Disord.

